# Genome-wide association study of *Striga* resistance in early maturing white tropical maize inbred lines

**DOI:** 10.1186/s12870-020-02360-0

**Published:** 2020-05-11

**Authors:** Samuel Adeyemi Adewale, Baffour Badu-Apraku, Richard Olutayo Akinwale, Agre Angelot Paterne, Melaku Gedil, Ana Luísa Garcia-Oliveira

**Affiliations:** 1 International Institute of Tropical Agriculture (IITA), PMB 5320, Oyo Road, Ibadan, Nigeria; 2 Department of Crop Production and Protection, Obafemi Awolowo University, Ile-Ife, Nigeria

**Keywords:** DArTseq markers, Genome-wide association study, Marker-assisted selection, *Striga hermonthica*, *Striga* resistance, *Zea mays* L

## Abstract

**Background:**

*Striga hermonthica* (Benth.) parasitism militates against increased maize production and productivity in savannas of sub-Saharan Africa (SSA). Identification of *Striga* resistance genes is important in developing genotypes with durable resistance. So far, there is only one report on the existence of QTL for *Striga* resistance on chromosome 6 of maize. The objective of this study was to identify genomic regions significantly associated with grain yield and other agronomic traits under artificial *Striga* field infestation. A panel of 132 early-maturing maize inbreds were phenotyped for key agronomic traits under *Striga*-infested and *Striga*-free conditions. The inbred lines were also genotyped using 47,440 DArTseq markers from which 7224 markers were retained for population structure analysis and genome-wide association study (GWAS).

**Results:**

The inbred lines were grouped into two major clusters based on structure analysis as well as the neighbor-joining hierarchical clustering. A total of 24 SNPs significantly associated with grain yield, *Striga* damage at 8 and 10 weeks after planting (WAP), ears per plant and ear aspect under *Striga* infestation were detected. Under *Striga*-free conditions, 11 SNPs significantly associated with grain yield, number of ears per plant and ear aspect were identified. Three markers physically located close to the putative genes GRMZM2G164743 (bin 10.05), GRMZM2G060216 (bin 3.06) and GRMZM2G103085 (bin 5.07) were detected, linked to grain yield, *Striga* damage at 8 and 10 WAP and number of ears per plant under *Striga* infestation, explaining 9 to 42% of the phenotypic variance. Furthermore, the S9_154,978,426 locus on chromosome 9 was found at 2.61 Mb close to the *ZmCCD1* gene known to be associated with the reduction of strigolactone production in the maize roots.

**Conclusions:**

Presented in this study is the first report of the identification of significant loci on chromosomes 9 and 10 of maize that are closely linked to *ZmCCD1* and *amt5* genes, respectively and may be related to plant defense mechanisms against *Striga* parasitism. After validation, the identified loci could be targets for breeders for marker-assisted selection (MAS) to accelerate genetic enhancement of maize for *Striga* resistance in the tropics, particularly in SSA, where the parasitic weed is endemic.

## Background

*Striga hermonthica* is a parasitic weed causing remarkable reduction in maize yield in the SSA and threatens the livelihoods of more than 300 million people [1]. *Striga* infestation is most severe in areas with low soil fertility, low rainfall and farming systems characterized by poor crop management practices and little use of inputs such as fertilizer, pesticides, and improved seeds [2]. Yield losses attributable to *Striga* parasitism range from 20 to 80% and the levels of infestation are often so high that maize may suffer 100% yield loss, and farmers could be forced to abandon their fields [3–6].

*Striga* seeds germinate in response to strigolactones in the root exudates of maize plants. Germinated seeds develop haustoria which attach to the roots of the host plants through which photosynthates and nutrients are transferred from the maize to the *Striga* plant [1]. Once attached to maize roots, the *Striga* plants survive by siphoning-off water and nutrients from the host plant for its own growth and development. The development of *Striga* plants impairs the normal host-plant growth, resulting in a large reduction in plant height, biomass, and eventually grain yield [7]. Feasible control options include intercropping, rotation of cereals with crops that are not susceptible to *S. hermonthica* such as cotton (*Gossypium* spp), soybean (*Glycine max L.*), and cowpea (*Vigna unguiculata L.*), herbicide treatment, hand-pulling, use of catch and trap crops, high nitrogen fertilization and use of tolerant and resistant varieties [1]. Among these options, host plant resistance is the most economical, sustainable and environmentally friendly.

During the past two decades, the maize improvement program of the International Institute of Tropical Agriculture (MIP-IITA) has placed a major emphasis on the development of stable and durable resistance to *Striga* from the wild maize (*Zea diploperennis L.*) and African landraces. Through these efforts, several populations, inbred lines and hybrids with durable *Striga* resistance have been developed. For example, the early-maturing *Striga* resistant and drought-tolerant maize inbred line, TZdEI 352, derived from a cross between the normal endosperm white maize population TZEW Pop DT STR and the *Z. diploperennis,* has displayed increased grain yield and durable *Striga* resistance [8]. Akaogu et al. [8] reported dominance gene effects to be higher than the additive effects for the number of emerged *Striga* plants in TZdEI 352, implying that non-additive gene action conditioned inheritance of *Striga* resistance. This inbred TZdEI 352 and other inbreds including TZEI 1203, TZEI 1252, and TZEI 1348, have been reported to possess significant positive/negative general combining ability (GCA) effects for grain yield, *Striga* damage, number of emerged *Striga* plants, ears per plant and ear aspect.

The present focus of the IITA-MIP is to transfer novel *Striga* resistance genes into *Striga* susceptible but outstanding genotypes, through gene stacking. This will allow the development of inbreds, hybrids and other available maize germplasm with durable resistance and increased maize production and productivity in SSA. With the advent of rapid genome-wide high-density marker data using high-throughput and next-generation sequencing technologies, GWAS has become a common tool for identifying resistance genes and/or loci through genome-wide association mapping [9, 10]. Identified genes and/or loci, once validated, could be fixed to provide maize germplasm with durable *Striga* resistance in tropical maize.

Information on the identification of QTL for *Striga* resistance in maize is very limited. Amusan [11] mapped two putative QTL on chromosome 6 of maize, from an F_2_ mapping population involving a cross between the *Striga* susceptible inbred line, 5057 and the resistant inbred line ZD05. These two QTL accounted for 55% of the phenotypic variability with dominant effects overlapping in importance. Unlike maize, the progress in the identification of QTL for marker assisted selection in sorghum is more advanced. The identification of lg gene mutant alleles at the LGS1 (Low Germination Stimulant 1) locus on chromosome 5 of sorghum has reduced greatly the *Striga hermonthica* germination stimulant activity [12]. This gene was found to code for a sulfo-transferase enzyme, and when silenced led to a change of 5-deoxystrigol into orobanchol compounds in the root exudates [12]. In addition, other loci have been reported to play important roles in parasitic resistance, including the genes *CCD1*, *CCD7* and *CCD8* [13, 14]. In maize, roots with mycorrhizal formations have shown a higher *ZmCCD1* expression and induced lower germination of *Striga* [13]. Liu et al. [15] provided evidence for strigolactones and strigolactone perception genes of the MAX-2-type in *Striga hermonthica*, namely *ShCCD7* and *ShCCD8*. In tobacco, the silencing of *CCD7* and *CCD8* genes retarded the virus parasite formation in the host, indicating that these two genes are key in parasitic life cycle [14].

In *Striga* resistance breeding, the primary traits of interest in selecting for resistance and high grain yield are host plant damage, number of emerged *Striga* plants, ears per plant and ear aspect [1, 16–18]. The host plant damage is positively correlated with the number of emerged *Striga* plants, and the two traits are negatively correlated with yield. Therefore, there is need to identify the genomic regions and genes that control the inheritance of grain yield and closely associated traits for successful use of MAS in maize improvement under *Striga* field infestation. Identification of genomic regions for *Striga* resistance would therefore facilitate rapid and efficient transfer of resistance genes to susceptible maize genotypes. The objectives of this study were to i) determine the genetic structure of a panel of 132 diverse early maturing white maize inbred lines with varying levels of resistance to *Striga hermonthica* parasitism and ii) identify putative genes associated with grain yield and other *Striga* adaptive traits under *Striga-*infested and *Striga-*free environments, using GWAS.

## Results

### Evaluation of phenotypic traits

Analysis of variance (ANOVA) across *Striga* infested environments revealed significant genotype (G) and environment (E) mean squares for all measured traits except environment mean squares for grain yield and *Striga* damage at 8 WAP (Table 1). Significant G x E mean squares were observed for ears per plant and number of emerged *Striga* plants at 8 and 10 WAP. Broad sense heritability (H^2^) estimates under artificial *Striga* infestation ranged from 47% for number of emerged *Striga* plants at 10 WAP to 71% for *Striga* damage at 10 WAP. Moderately high heritability estimates ≥60% were recorded for the *Striga* resistance indicator traits (grain yield, ears per plant, ear aspect, *Striga* damage at 8 and 10 WAP, and number of emerged *Striga* plants at 8 WAP) under *Striga* infestation. Under *Striga*-free conditions, the combined ANOVA displayed significant G, E and G x E mean squares for grain yield, ears per plant and ear aspect. Broad sense heritability estimates ranged from 28% for ears per plant to 58% for ear aspect when *Striga*-free.

**Table 1:** Mean squares from the analysis of variance of grain yield and other agronomic traits of 132 tropical early maturing maize inbred lines evaluated under *Striga*-infested and *Striga*-free environments, at Mokwa between 2017 and 2018

Source of variation	Df	Yield, kg/ha	Ears per plant	Ear aspect	*Striga* damage rating at 8 WAP	*Striga* damage rating at 10 WAP	Emerged *Striga* plants at 8 WAP	Emerged *Striga* plants at 10 WAP
*Striga-*infested conditions								
Environment (E)	1	1,041,553	4.32**	11.49**	0.29	44.16**	12.81**	19.58**
Block (Rep*E)	44	493501*	0.03	1.23	0.98	1.17*	0.15	0.09
Rep (E)	2	1117085*	0.04	3.64*	0.36	0.58	0.66**	0.10
Genotype (G)	131	846079**	0.05**	2.24**	2.08**	2.65**	0.34**	0.26**
Env*Genotype	131	320,753	0.05**	0.92	0.71	0.84	0.14*	0.15**
Error	218	323,545	0.02	0.97	0.70	0.73	0.11	0.09
Heritability		0.63	0.61	0.60	0.67	0.71	0.60	0.47
*Striga*-free conditions								
Environment (E)	1	1484892*	4.13**	120.27**				
Block (Rep*E)	44	352,004	0.06	0.83				
Rep (E)	2	3428522**	0.35**	5.38**				
Genotype (G)	131	1059290**	0.15**	3.10**				
Env* Genotype	131	495955**	0.10**	1.28**				
Error	218	347,667	0.07	0.73				
Heritability		0.54	0.28	0.58				

**Fig. 1:**
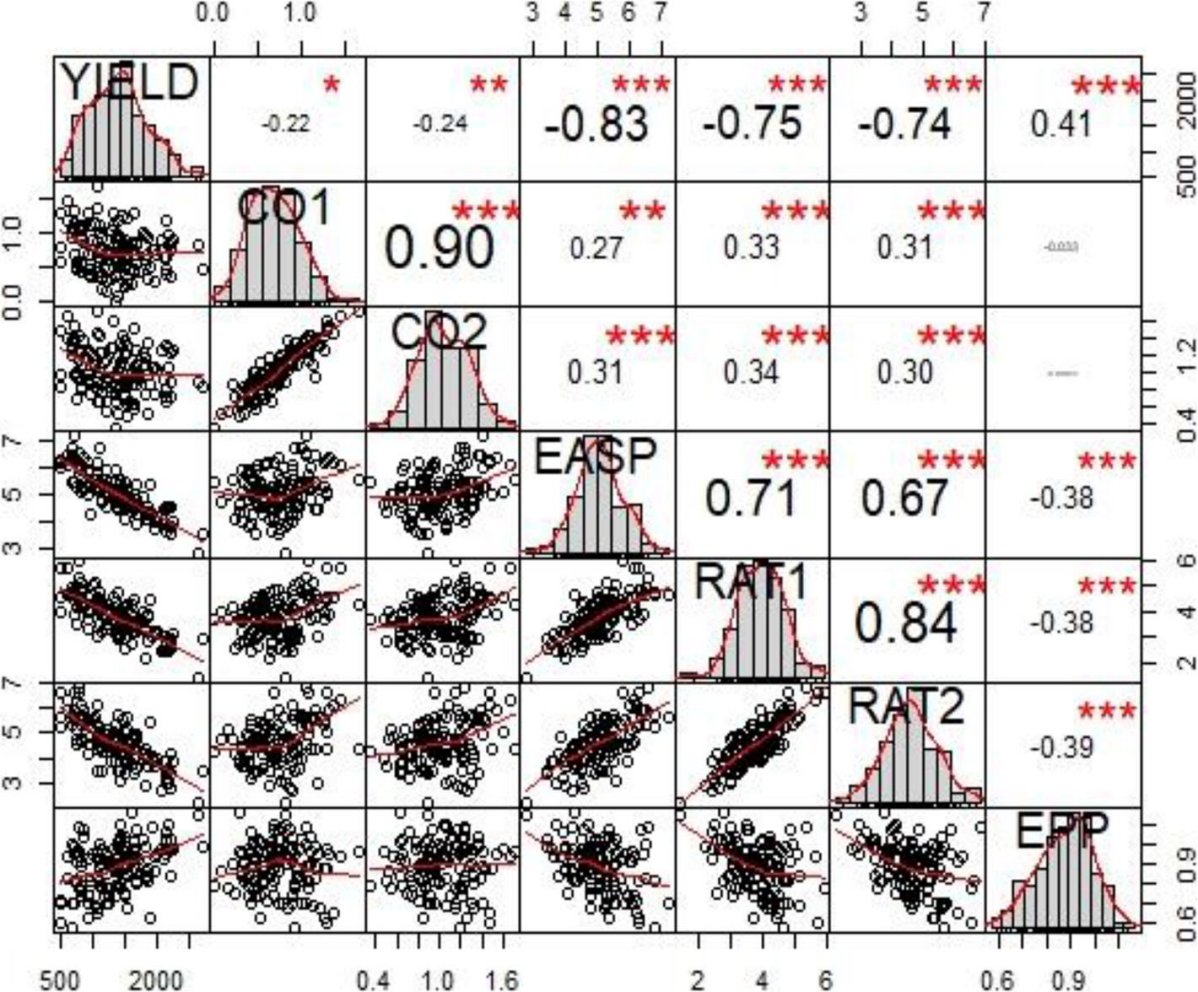
Correlation coefficients between *Striga* resistance indicator traits and other agronomic traits of early maturing maize inbred lines under artificial *Striga* infestation at Mokwa between 2017 and 2018. YIELD = grain yield, CO1 = number of emerged *Striga* plants at 8 WAP, CO2 = number of emerged *Striga* plants at 10 WAP, EASP - ear aspect, RAT1 = *Striga* damage symptoms rating at 8 WAP, RAT2 - *Striga* damage symptoms rating at 10 WAP, EPP - number of ears per plant

The phenotypic correlations among grain yield and other measured *Striga* adaptive traits differed under artificial *Striga* infestation (Fig. 1). Grain yield had significant negative correlation with ear aspect (*r* = − 0.83**), *Striga* damage at 8 WAP (*r* = − 0.75**) and 10 WAP (*r* = − 0.74**), number of emerged *Striga* plants at 8 WAP (*r* = − 0.22*) and 10 WAP (*r* = − 0.24**), and significant positive correlation with ears per plant (*r* = 0.41**). Similarly, significant positive correlations were obtained between *Striga* damage at 8 WAP and *Striga* damage at 10 WAP (*r* = 0.84**), number of emerged *Striga* plants at 8 WAP and number of emerged *Striga* plants at 10 WAP (*r* = 0.90**), ear aspect and *Striga* damage (*r* = 0.71**).

### Population structure and genetic diversity

A total of 47,440 SNPs were generated for the maize inbred lines using DArT sequencing technology. After quality filtering of the unmapped and multilocation markers, SNPs with missing values > 10%, heterozygosity > 20% and MAF < 5% were excluded and thus, a total of 7224 SNPs were retained for the analysis (Additional file 1: Figure S1). The results of the summary statistics of the maize inbred lines based on the filtered 7224 markers are displayed in Additional file 2: Table S1. The PIC ranged from 0.09 to 0.37 with an average of 0.26 whereas the heterozygosity averaged 0.07 and varied from 0.00 to 0.20. The minor allele frequencies of the 7224 primers recorded a mean of 0.24 with minimum and maximum minor allele frequencies of 0.05 and 0.5, respectively. Gene diversity varied from 0.10 to 0.50 with an average of 0.33.

**Fig. 2:**
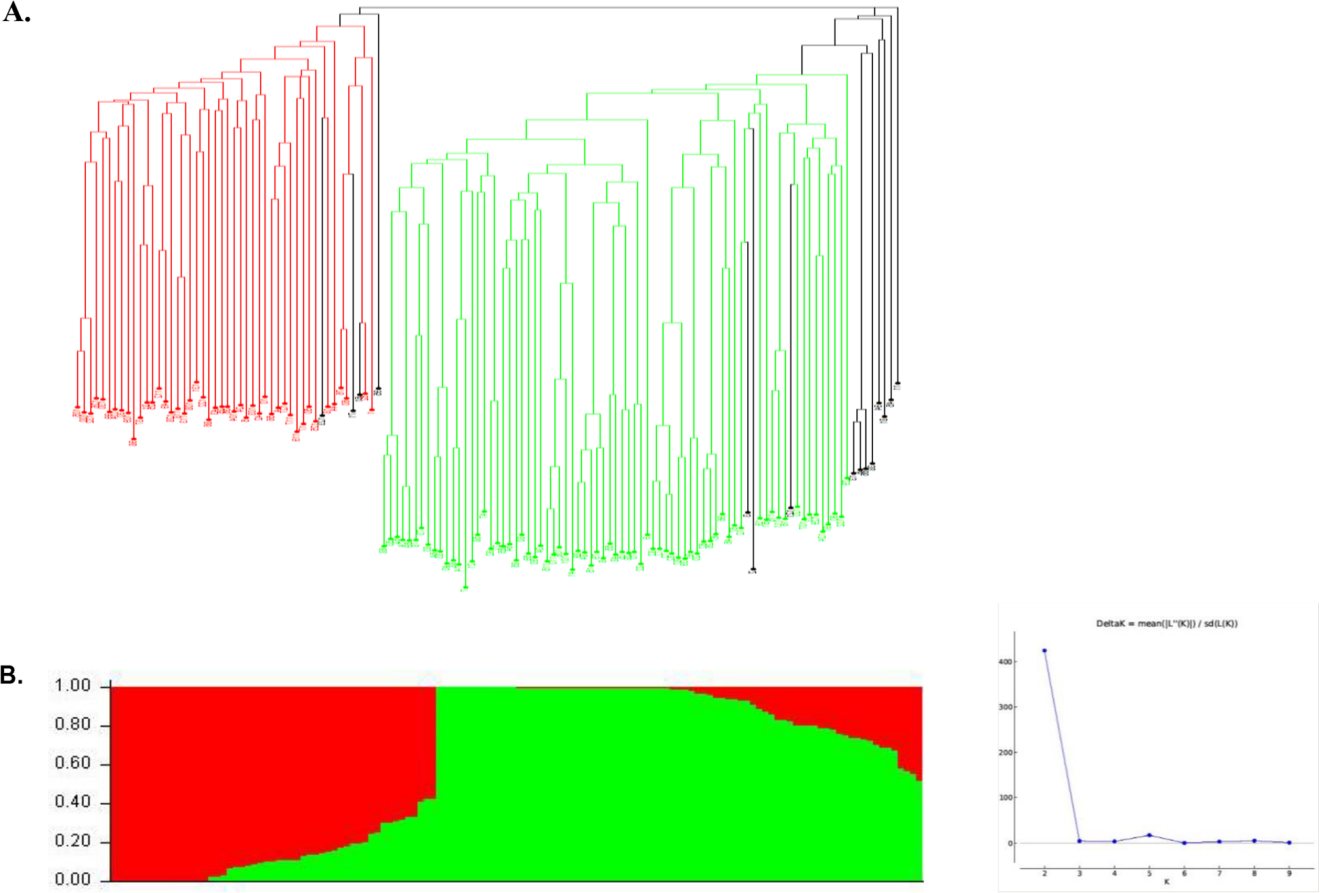
(**a**) Neighbor-joining tree displaying the genetic relationship among the 132 maize inbred lines based on 7224 DArTseq markers. Inbred lines are colour-coded according to the populations’ substructure assignment to clusters based on STRUCTURE results (**b**). Analysis of the population structure of the 132 maize panel based on 7224 DArTseq markers using STRUCTURE at K = 2

Population structure was inferred through the admixture model-based clustering method [19]. The STRUCTURE algorithm and STRUCTURE HARVESTER results revealed delta K plot peak value of two (Fig. 2b). The results of the unweighted neighbor joining phylogenetic tree, color coded from the STRUCTURE results, also indicated two major groups (Fig. 2a). At k = 2, 89% of the inbred lines were assigned into two groups, and only 11% of the lines were assigned in the mixed group. A total of 45 inbred lines were placed in group 1, 72 in group 2 and 15 in the mixed group. Each group comprised inbred lines from two or more germplasm sources. Of the 45 inbred lines placed in group 1, 26 were derived from the biparental crosses (TZE COMP 5-W DT C7 x TZEI 56, TZE COMP 5-W DT C7 x TZEI 65, TZE COMP 5-W DT C7 x TZEI 18), 8 were obtained from TZE COMP 5-W DT C7 x TZEI 31 while 6 were extracted from TZE COMP 5-W DT C7 x TZEI 87, TZE COMP 5-W DT C7 x TZEI 2. Furthermore, the inbred testers TZEI 1 and TZEI 18 (derived from population TZE-W Pop STR Co) as well as TZEI 7, TZEI 31 and TZdEI 352 (derived from WEC STR S7, TZE-W Pop x LD and TZE-W Pop STR 107, respectively), were also classified into group 1. Of the 72 inbred lines classified into group 2, 36 were derived from the biparental crosses (TZE COMP 5-W DT C7 x TZEI 56, TZE COMP 5-W DT C7 x TZEI 65, TZE COMP 5-W DT C7 x TZEI 18), 28 derived from TZE COMP 5-W DT C7 x TZEI 87, TZE COMP 5-W DT C7 x TZEI 2 and 7 from TZE COMP 5-W DT C7 x TZEI 31. In addition, the inbred tester TZEI 19 developed from TZE-W Pop STR Co was placed in group 2. The inbred lines placed in the two groups had in common the broad based *Striga* resistant population TZE COMP 5-W DT C7. However, the grouping of the inbred lines was largely based on the reactions of the inbred lines to *Striga* as each group contained both resistant and susceptible inbred lines. Using the IITA selection base index, 38 of the 45 inbred lines placed in group 1 were *Striga* resistant while 7 were susceptible (Table not shown). In contrast, out of 72 inbred lines placed in group 2, there were 52 each of *Striga* resistant and 20 susceptible inbreds. It is interesting that the grouping of most of the inbred testers was largely based on resistance to *Striga*. For example, the *Striga* resistant inbred testers TZdEI 352, TZEI 7 and TZEI 1, the moderately resistant testers TZEI 18, and susceptible tester TZEI 31 were placed in group 1 while the *Striga* susceptible inbred tester TZEI 19 was placed in group 2.

### Population linkage disequilibrium (LD)

Linkage disequilibrium analysis revealed the presence of 359,926 loci pairs within a physical distance extending up to 55,266,364 bp. About 22.05% (79,364) of the loci pairs were in significant LD (*P* < 0.001). In addition, 1231 (0.30%) of the loci pairs were in complete LD (R^2^ = 1). Pearson’s correlation coefficients showed negative and significant correlation (*r* = − 0.035) between the linkage disequilibrium (R^2^) and the physical distance (bp) as well as between the *P*-value and R^2^ (*r* = − 0.59), indicating the existence of linkage decay. The rate of LD decay differed across the chromosomes (Additional file 3: Figure S2), ranging from 6948 kb for chromosome 5 to 8367 kb for chromosome 10 at r^2^ < 0.2 (Table 2). The average pairwise r^2^ and LD decay at r^2^ < 0.2 for the entire genome was approximately 0.03 and 7636 kb, respectively. The slowest LD decay was observed for chromosome 10 (8367 kb), followed by chromosome 4 (8213 kb) and chromosome 8 (7924 kb).

**Table 2:** Details of LD decay distance observed at R^2^ < 0.2 on the different chromosomes and the entire maize genome

Chromosome	Size (Mb)	Mean r^2^	LD decay distance (Kb) at r^2^ < 0.2
1	167.12	0.04	6971.163
2	117.22	0.04	7178.097
3	140.09	0.03	7531.945
4	141.96	0.03	8212.815
5	105.37	0.04	6947.642
6	110.94	0.04	7893.009
7	113.14	0.03	7921.105
8	113.19	0.03	7923.937
9	95.80	0.03	7412.867
10	91.09	0.03	8367.483
Whole genome	123.45	0.40	7542.275
Average	119.59	0.03	7636.006

### Genome-wide association and LD analysis

Under artificial *Striga* infestation, 24 significant SNPs were detected for five different traits at a GWAS threshold of –log (p) = 4 (Table 3). The trait variation explained by each marker R^2^ varied from 9 to 42%. Of the 24 SNPs that were significant, nine were located on chromosome 10. Three markers located on chromosomes 10 and 9 were associated with grain yield and explained about 34% of the phenotypic variation. Ears per plant was associated with seven markers located on chromosomes 4, 5, 7, and 10. These markers revealed 9 to 13% of the phenotypic variation. Ear aspect had only one significant SNP located on chromosome 10 under *Striga*-infestation. Furthermore, five SNPs located on chromosomes 3, 7, 8 and 10 were detected for *Striga*-damage at 8 WAP, 7 SNPs on chromosomes 1, 3, 9 and 10 for *Striga*-damage at 10 WAP and one SNP on chromosome 1 for number of emerged *Striga* plants at 8 WAP. The marker S10_133,224,759 explained the highest proportion of the phenotypic variance (42%) while S5_215,584,703 and S4_76,136,186 explained the least proportion (9%) of the phenotypic variation. Marker S10_133,224,759 located on chromosome 10 was found associated repeatedly with grain yield and *Striga* damage at 8 and 10 WAP. Similarly, marker S10_112,661,466 on chromosome 10 was found to be common for *Striga* damage at 8 and 10 WAP.

**Table 3:** DArTseq markers having significant association with *Striga*-adaptive traits of 132 inbred lines evaluated under *Striga*-infested and *Striga*-free conditions at Mokwa across years 2017 and 2018

Trait	SNP	Chr	Position	P-value	Marker R^2^
*Striga*-infested conditions					
Grain yield	S10_96,965,850	10	96,965,850	4.95 × 10^−4^	0.343
	S9_7,249,203	9	7,249,203	5.25 × 10^−4^	0.342
	S10_133,224,759	10	133,224,759	8.43 × 10^−4^	0.336
Ears per plant	S5_207,493,972	5	207,493,972	1.04 × 10^−4^	0.130
	S7_137,739,978	7	137,739,978	1.21 × 10^− 4^	0.127
	S10_16,561,232	10	16,561,232	2.16 × 10^−4^	0.118
	S7_140,475,074	7	140,475,074	6.08 × 10^−4^	0.101
	S10_16,804,228	10	16,804,228	8.98 × 10^−4^	0.095
	S5_215,584,703	5	215,584,703	9.44 × 10^−4^	0.094
	S4_76,136,186	4	76,136,186	9.81 × 10^−4^	0.094
*Striga* damage at 8 WAP	S10_133,224,759	10	133,224,759	4.41 × 10^−5^	0.420
	S7_79,624,222	7	79,624,222	5.14 × 10^−4^	0.393
	S8_52,394,249	8	52,394,249	5.94 × 10^−4^	0.391
	S10_112,661,466	10	112,661,466	8.01 × 10^−4^	0.388
	S3_179,448,461	3	179,448,461	9.91 × 10^−4^	0.385
*Striga* damage at 10 WAP	S10_133,224,759	10	133,224,759	3.55 × 10^−5^	0.333
	S10_112,661,466	10	112,661,466	1.88 × 10^−4^	0.311
	S3_179,448,461	3	179,448,461	2.61 × 10^−4^	0.307
	S1_9,730,753	1	9,730,753	3.75 × 10^−4^	0.303
	S3_47,343,213	3	47,343,213	4.32 × 10^−4^	0.301
	S3_143,135,181	3	143,135,181	4.60 × 10^−4^	0.300
	S9_154,978,426	9	154,978,426	8.99 × 10^−4^	0.292
*Striga* count at 8 WAP	S1_102,219,766	1	102,219,766	3.42 × 10^−4^	0.221
Ear aspect	S10_144,129,548	10	144,129,548	5.94 × 10^−4^	0.258
*Striga*-free conditions					
Grain yield	S4_177,968,538	4	177,968,538	8.09 × 10^−4^	0.148
	S3_188,682,443	3	188,682,443	9.12 × 10^−4^	0.141
Ears per plant	S2_210,201,646	2	210,201,646	6.86 × 10^−4^	0.143
	S8_145,372,017	8	145,372,017	7.55 × 10^−4^	0.132
	S1_67,606,758	1	67,606,758	8.57 × 10^−4^	0.130
	S1_68,549,674	1	68,549,674	9.07 × 10^−4^	0.129
Ear aspect	S8_96,677,591	8	96,677,591	4.00 × 10^−4^	0.207
	S6_97,507,107	6	97,507,107	5.74 × 10^−4^	0.235
	S3_176,205,016	3	176,205,016	5.57 × 10^−4^	0.203
	S8_161,625,705	8	161,625,705	6.00 × 10^−4^	0.201
	S6_108,391,162	6	108,391,162	9.98 × 10^−4^	0.194

**Fig. 3:**
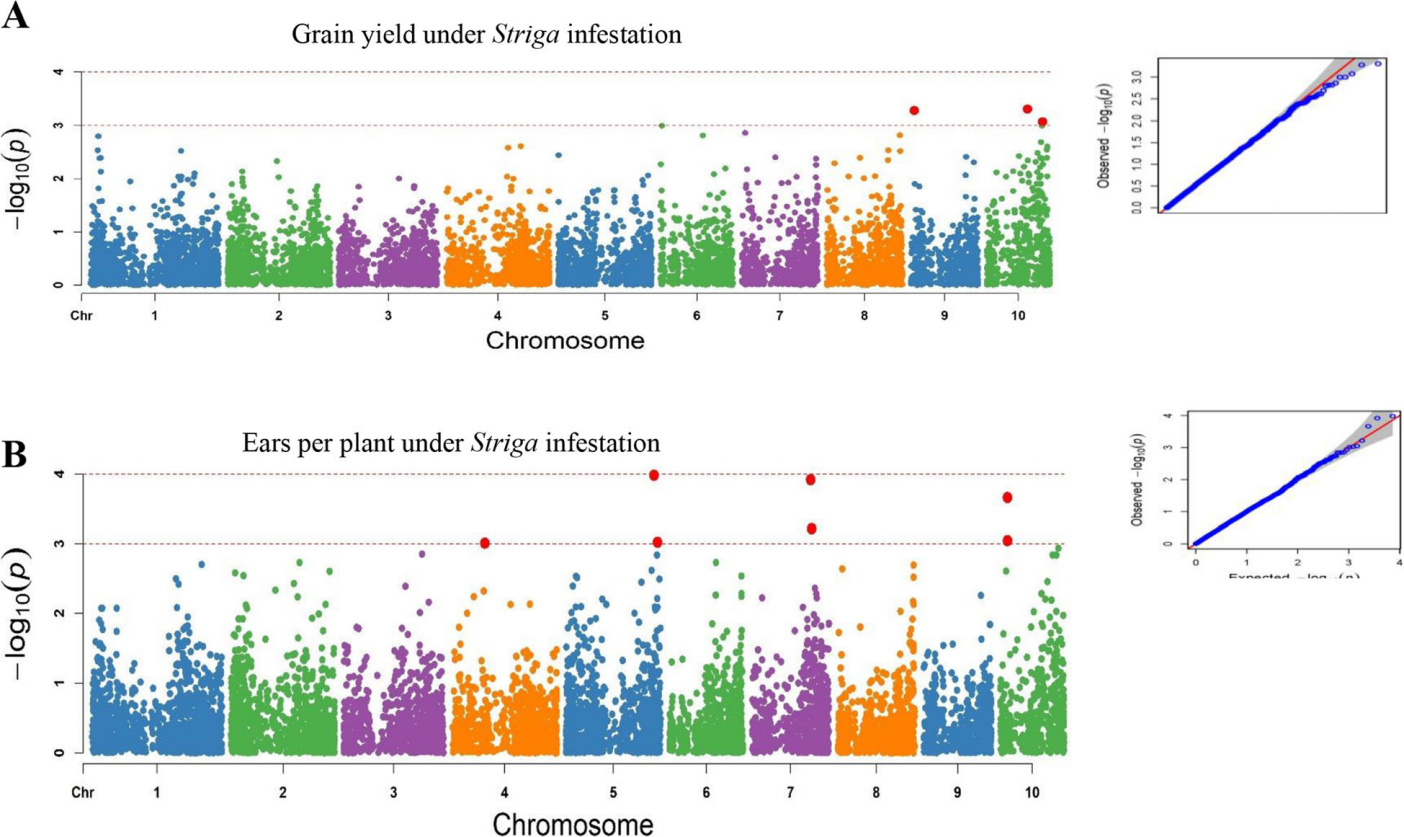
The Manhattan and Q-Q plots of the SNP-based associations mapping for grain yield and ears per plant under artificial *Striga* infestation

**Fig. 4:**
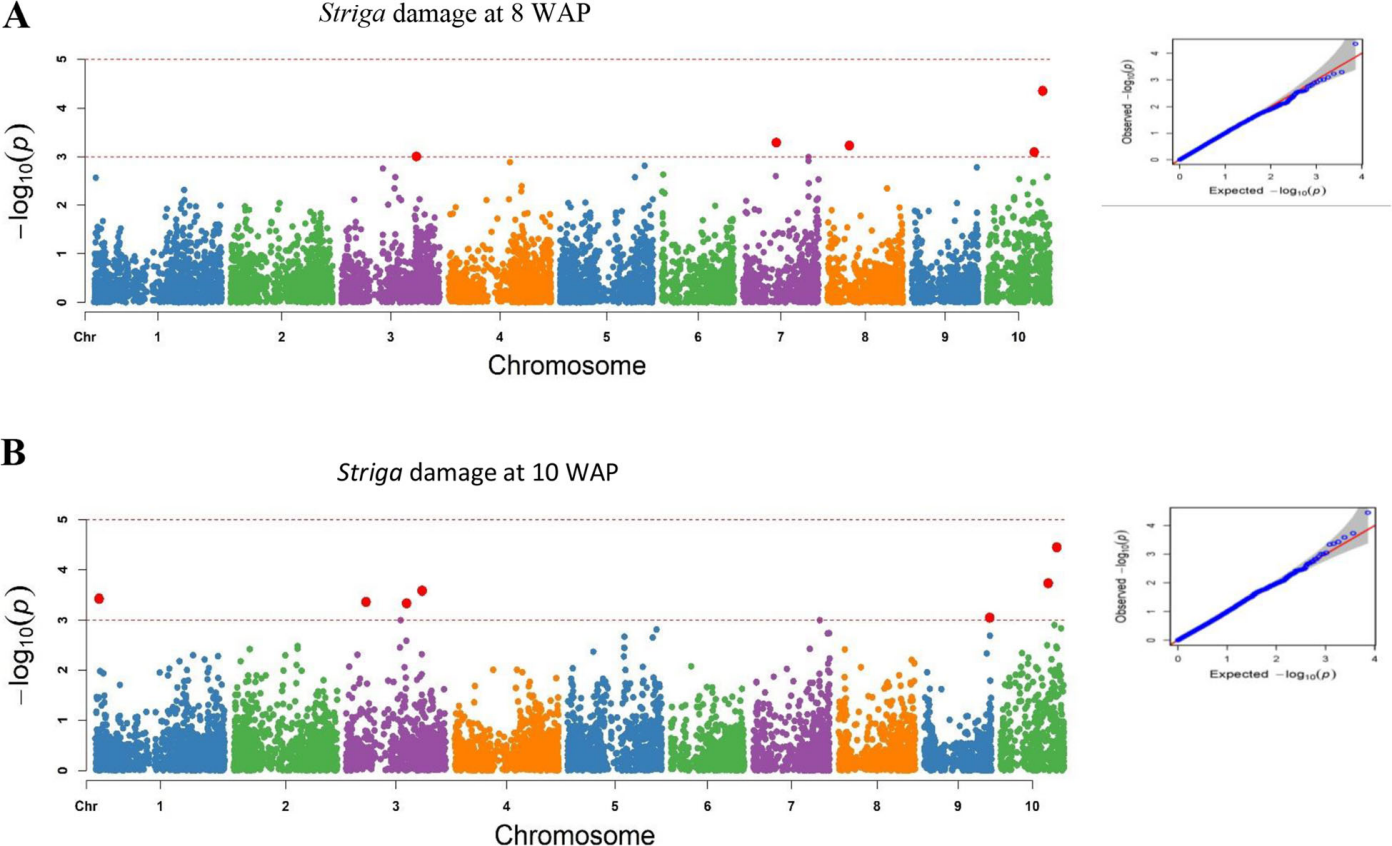
The Manhattan and Q-Q plots of the SNP-based associations mapping for *Striga* damage at 8 WAP and 10 WAP under artificial *Striga* infestation

**Fig. 5:**
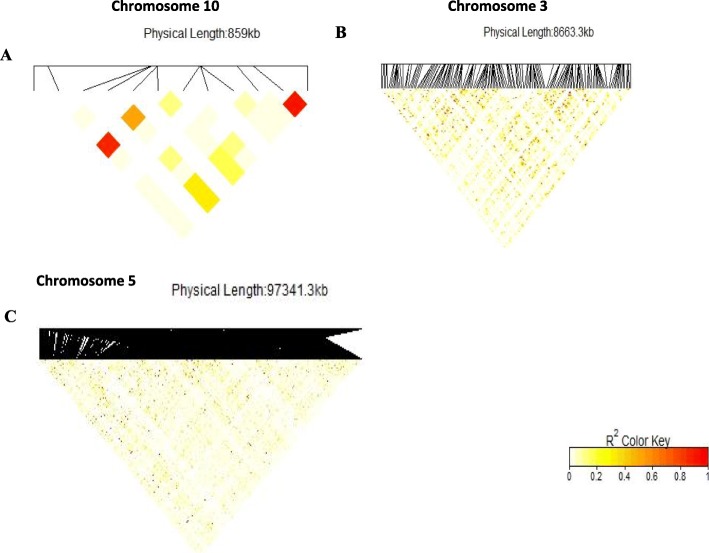
Local LD block surrounding (**a**) *amt5* gene on chromosome 10. (**b**) ereb13 gene on chromosome 3. (**c**) *lg2* gene on chromosome 3. The square lattice panel represents the extent of LD based on r^2^. The R^2^ color key indicates the degree of significant association

Under *Striga*-free conditions, 11 SNPs significantly associated with grain yield, ears per plant and ear aspect were detected (threshold of –log (p) = 4), accounting for 13 to 23% of the total phenotypic variation observed among the traits. Two markers located on chromosomes 3 and 4 were associated with grain yield and explained 14 to 15% of the phenotypic variation in grain yield. Number of ears pe*r* plant had four significant SNPs situated on chromosomes 8, 2 and 1 explaining 13 to 21% of the phenotypic variation. In addition, five different SNPs located on chromosomes 3, 6 and 8 were associated with ear aspect, describing 19 to 24% of the phenotypic variation. Significant SNPs identified for grain yield, ears per plant and ear aspect under *Striga*-infestation were different from those under *Striga*-free conditions. The results of the SNPs for grain yield, ears per plant and *Striga* damage under *Striga* infestation are illustrated in the Manhattan and quantile-quantile plots (Figs. 3 and 4). The quantile-quantile plots revealed good data adjustment and a few significant SNPs above the interval for the expected values of the null hypothesis. LD block heatmaps of the three candidate gene loci identified are shown in Fig. 5. LD analysis of each of the three loci revealed that these markers had relatively low LD parameter (R^2^), indicating relatively low correlation with each other.

### Candidate loci for *Striga hermonthica* resistance

The genomic regions of the significant SNPs were examined to identify the protein-coding genes located in or close to the significant SNPs based on the data retrieved from the maize genetic database (http://www.maizegdb.org/). The list of annotated genes, including those encoding uncharacterized proteins of the most significant SNPs are presented in Table 4. The SNP S10_133,224,759 having significant association with grain yield and *Striga* damage under *Striga* infestation was located within the candidate gene *amt5* (ammonium transporter 5) with identifier GRMZM2G164743. Similarly, SNP S10_16,561,232 having strong association with ears per plant under *Striga* infestation was located on another candidate gene GRMZM2G310674, which encodes a Polynucleotidyl transferase putative protein, belonging to the ribonuclease H-like superfamily protein. On chromosome 9, SNP S9_7,249,203 associated with grain yield under *Striga* infestation was physically located within the putative genes GRMZM2G080044 and GRMZM5G898880. These genes are responsible for cellular respiration, oxidative phosphorylation and NAD (P) H dehydrogenase activities. Additionally, SNP S9_154,978,426 significantly associated with *Striga* damage was located on the protein-coding putative gene GRMZM2G010017 that encodes a protein phosphatase 2C family protein and 2.62 Mb close to the *Zmccd1* gene, GRMZM2G057243. On chromosome 5, S5_207,493,972 and S5_215,584,703 associated with ears per plant under *Striga* infestation were located on the GRMZM2G315127 and 131.43 kb close to GRMZM2G103085 putative genes, respectively. The putative gene GRMZM2G315127 encodes for an uncharacterized protein while the GRMZM2G103085/EREB139 putative gene encodes for ethylene-responsive element-binding proteins. Marker S3_179,448,461 having significant association with *Striga* damage was found 60.7 kb close to the candidate gene *lg2,* GRMZM2G060216, which encodes a basic leucine zipper protein transcription factor. On chromosome 7, S7_137,739,978 having significant association with ears per plant under *Striga* infestation was located on the putative gene GRMZM2G016836, which encodes the NAD (P)-binding Rossmann-fold superfamily protein.

**Table 4:** Candidate genes for each significant SNP associated with *Striga* adaptive traits under *Striga* infestation and *Striga-*free conditions

Trait	Chr	Position	Gene ID	Encoding products
*Striga*-infested conditions				
Grain yield and *Striga* damage	10	133,224,759	GRMZM2G164743	*amt5* (ammonium transporter protein)
Grain yield	9	7,249,203	GRMZM2G080044, GRMZM5G898880	Cellular respiration, oxidative phosphorylation, alternative NAD (P) H dehydrogenase activities.
*Striga* damage	9	154,978,426	GRMZM2G057243	*ZmCCD1* gene
			GRMZM2G010017	Protein phosphatase 2C family protein
	3	179,448,461	GRMZM2G060216	*lg2* (basic leucine zipper protein transcription factor)
Ears per plant	10	16,561,232	GRMZM2G310674	Polynucleotidyl transferase, ribonuclease H-like superfamily protein
	7	137,739,978	GRMZM2G016836	NAD (P)-binding Rossmann-fold superfamily protein
	5	207,493,972	GRMZM2G315127	Plant protein of unknown function
	5	215,584,703	EREB139, GRMZM2G103085	AP2-EREBP (ethylene-responsive element-binding proteins)
*Striga*-free conditions				
Ears per plant	2	210,201,646	GRMZM2G009412, GRMZM2G125527	Zinc-binding ribosomal protein family protein
	1	67,606,758	GRMZM2G078806	Putative uncharacterized protein
	1	68,549,674	GRMZM2G125314	LOL3 (protein degradation)
Ear aspect	8	96,677,591	GRMZM2G083344	Glucose-6-phosphate/phosphate translocator-related

Under *Striga*-free conditions, S1_67,606,758 and S1_68,549,674 linked with ears per plant on chromosome 1 were found on the GRMZM2G078806 and GRMZM2G125314 putative genes, respectively. These genes encode for a putative uncharacterized protein and LOL3 (protein degradation), respectively. Similarly, SNP S2_210,201,646 on chromosome 2 associated with ears per plant was located on the putative genes GRMZM2G009412 and GRMZM2G125527. These genes encode for Zinc-binding ribosomal protein. SNP S8_96,677,591 significantly linked to ear aspect was found within the gene GRMZM2G083344, encoding glucose-6-phosphate/phosphate translocator-related proteins.

## Discussion

The significant variation observed among the inbred lines for grain yield and other agronomic traits revealed the existence of adequate genetic variability among the early maturing maize inbred lines under *Striga*-infested and *Striga*-free research conditions. The significant environment mean squares observed for most traits in the present study showed the distinctness of the environments in discriminating among the genotypes under each research condition [20, 21]. Moderate to high heritability estimates detected for grain yield and other *Striga* adaptive traits implied increased power of SNP detection in the maize panel allowing for identification of true associations between a marker and putative gene [22–24]. The significant positive correlation observed between grain yield and ears per plant and significant negative correlations between grain yield and *Striga* damage, number of emerged *Striga* plants and ear aspect suggested simultaneous improvement of these traits would result in high yield under *Striga* infestation. Previous studies identified grain yield, number of emerged *Striga* plants, *Striga* damage, ears per plant and ear aspect as the most reliable traits in selecting for *Striga* resistant maize genotypes, thus justifying their inclusion in the selection index for yield improvement in *Striga*-prone environments in SSA [17, 25].

Approximately 70% of the SNPs used in the study had heterozygosity < 5%. The lower values of heterozygosity observed among the inbred lines in the two groups indicated that the SNPs were efficient in forming homogenous groups. Thus, making them a valuable resource for genetic studies and association mapping where uniformity of inbred lines and genetic divergence are required [26]. The average PIC value of 0.26 obtained in this study is higher than that reported by Adu et al. [26] but comparable to those reported previously by Simko et al. [27] and Zhang et al. [28]. This reveals the informativeness of the markers used in this study. The frequency of minor alleles is a crucial factor influencing the accuracy of LD analysis and GWAS, especially when using small number of genotypes [29, 30]. The filtered high-quality SNPs used in this study had a large proportion of MAFs distributed uniformly across the genome, frequencies greater than 5%. Most of the significant SNPs identified in the present study had MAFs greater than 10%, implying the positive detection power of the GWAS as the biasness associated with rare alleles was removed [31].

Estimation of population structure and within-group relatedness in maize genomic association studies is necessary in order to reduce the risk of false-positives [22, 32]. The 132 inbred lines used in this study were classified into two major clusters (k = 2) by the DArTseq markers and each cluster was further partitioned into sub-clusters. Results obtained from the population structure analysis were similar to those previously reported by Cui et al. [33] in sesame, Campa et al. [34] in common bean, Mogga et al. [35] in rice, Maldonado et al. [36] in maize. It is interesting that the grouping of the inbred lines by the markers was mostly based on the reactions of the inbred lines to *Striga* as each group contained both resistant and susceptible inbred lines. The results of the population structure analysis were confirmed by the neighbor joining phylogenetic tree. The average genome-wide LD decay was estimated at 7636kbp at r^2^ < 0.2. Previous studies reported LD less than 1000 bp for maize landraces, about 100 kb for commercial elite breeding lines and about 830Kbp for diverse breeding lines [30, 37]. The existence of marker pairs in LD over long distances in the present study was expected since such large LD is a feature of advanced maize inbred lines that have gone through selection [30]. The large LD could lead to the identification of SNPs in genes that either cause or contribute to *Striga* resistance, or which act as linked markers associated with *Striga* resistance.

The model fitness for the GWAS was confirmed by inspecting quantile-quantile (QQ) plots that compared the observed and expected *p*-values under the null hypothesis of no associations. Our results revealed that majority of points in the QQ plots were aligned on the diagonal line for all the measured traits, indicating that spurious associations due to population structure and familial relatedness were largely corrected. Similar findings were reported by Kuki et al. [23], who identified genomic regions, including putative genes, associated with resistance to gray leaf spot disease in tropical maize under natural disease infection.

Marker-trait association analyses have demonstrated that association between specific phenotypes and genotypes within a genome, could lead to the discovery of genes controlling the traits [38]. In order to increase the level of resistance to *Striga* in the available early maturing tropical maize germplasm, 24 markers significantly associated with *Striga* damage, number of emerged *Striga* plants, number of ears per plant, ear aspect and grain yield under *Striga* infestation were identified, at the threshold of –log (p) = 4. These markers were located on chromosomes 10, 9, 8, 7, 5, 4, 3 and 1. In contrast to the present results, Amusan [11] identified two putative loci for resistance to *Striga* on chromosome 6 of maize, using SSR markers and composite interval mapping (CIM) in a late maturing maize F_2_ mapping population. The first QTL was found between markers umc2170 and bnlg1142 whereas the second QTL was found between SSR markers bnlg1867 and umc1014 [11]. These loci were found to govern the incompatible response to *Striga* parasitism and accounted for 55% of the phenotypic variation (PV) with predominance of dominance genetic effects over additive genetic effects in the expression of the two *Striga* resistance QTLs [11]. In our present study, no significant loci were identified on chromosome 6. The differences in the results of the two studies could be attributed to the differences in the genetic materials used in the two different studies. Furthermore, 11 markers were identified to be associated with grain yield, ears per plant and ear aspect under *Striga*-free conditions. These markers were located on chromosomes 8, 6, 4, 3 and 2. Lack of SNPs overlap for the measured traits under *Striga* infested and *Striga*-free environments indicated the genetic divergence between the two contrasting test environments. In addition, results of the present study showed that the *Striga* resistance indicator traits under artificial *Striga*-infested environments were complex in nature and were controlled by multiple minor QTLs with small effects distributed across the maize genome. The markers having significant association with *Striga* resistance indicator traits such as reduced *Striga* damage symptoms, increased number of ears per plant and high grain yield under *Striga* infestation could be used as candidate markers for simultaneous selection for the target traits in maize [1]. Allelic variations at each significant SNP were associated with 9 to 42% of the phenotypic variance, suggesting that these markers could be useful for marker-assisted selection for improved *Striga* resistance in tropical maize improvement programs. *Striga* resistance in maize is a polygenic trait making it relatively difficult to achieve good progress from selection. However, the identification of molecular markers tightly linked to functional genes is an important step towards development of genotypes with enhanced levels of resistance through gene pyramiding.

The most noticeable candidate genes identified in the present study are located at SNPs S9_154,978,426 and S10_133,224,759 on chromosomes 9 and 10, respectively. The SNP S9_154,978,426 is located at 2.62 Mb from the *ZmCCD1* gene on chromosome 9 [13]. It has been shown that maize roots colonized by arbuscular mycorrhizal fungi had a higher *ZmCCD1* expression that could limit *Striga* germination [13]. This finding is particularly interesting as it is well known that the lowering of strigolactone production is still the best-known mechanism for preventing *Striga* germination [39, 40]). Since the *ZmCCD1* gene is involved in the formation of the yellow pigment apocarotenoids [13], it would be interesting in the future to understand the carotenoid levels of the lines and its relationship with *Striga* resistance in the maize inbred lines. On chromosome 10, the SNPs S10_133,224,759 and S10_112,661,466 are particularly interesting because they are significant for *Striga* damage at 8 and 10 WAP whereas S10_133,224,759 was significant for grain yield under *Striga* infestation. The SNP S10_133,224,759 located at the physical coordinates chr 10: 133,224,759 had the largest proportion of phenotypic variance (46%) for *Striga* damage in the panel of the IITA early maturing maize inbred lines. This SNP marker is close to the functional gene GRMZM2G164743 (bin 10.05), which encodes an ammonium transporter protein (*amt5*). *AMT* genes have been identified in many plant species including *Zea mays* [41] and *Sorghum bicolor* [42]. Nitrate (NO^− 3^) and ammonium (NH^+ 4^) are the major forms of nitrogen (N) uptake in higher plants. The NH^+ 4^ ions accumulate in cells either by direct uptake from the rhizosphere via ammonium transporters (AMTs) or by reduction of NO^− 3^. Dechorgnat et al. [43] found *ZmAMT2.1* gene (a member of the AMT family) to be expressed in all organs with some specificity to roots and tassels. Interestingly, Koegel et al. [42] reported a similar expression pattern of the *ZmAMT2.1* orthologue in sorghum. The authors observed that in sorghum, *SbAMT2.1* was expressed in all organs studied with higher expression in roots and stamens. Nitrogen status of the plants is also closely associated with plant defense against *Striga* parasitism as several authors have reported significant reduction in number of emerged *Striga* plants under high nitrogen concentration [43, 44]. The SNP S10_133,224,759 indicated that the candidate gene (*amt5*) identified in the present study could be responsible for the defense mechanism against *Striga* parasitism in maize. The gene therefore could be a novel target for further unravelling of the regulatory mechanism of *Striga* resistance in the roots of maize plants and should be tested further in breeding programs for its usefulness in selecting inbred lines with resistance to *Striga hermonthica* parasitism.

Marked association around *lg2* gene was detected for *Striga* damage. The SNP S3_179,448,461, located at the long arm of chromosome 3, was found close (60.7 kb) to the candidate gene GRMZM2G060216 (3.06), which is associated with loci *lg2* (liguleless2). The *lg2* gene encodes a basic leucine zipper protein transcription factor and has mutants known to affect leaf angle in maize. In maize, *lg1* and *lg2* mutants have no ligule or auricle, leading to considerably more upright leaves than their normal counterparts, thereby increasing photosynthetic activity and eventually leading to significant grain yield increase in maize hybrids [45]. Thus, the *lg2* gene may be associated with maize plant defense mechanism under *Striga* infestation. Previous studies have revealed that *Striga* infection influences the rate of photosynthesis in the host plants’ leaves by decreasing the effectiveness of the photosynthetic process. This has been demonstrated in sorghum, millet, cowpea as well as in maize [7, 46–50]. The complex interplay between photosynthesis and plant defenses have been recently elucidated [51] and showed that both biological processes share common regulators. It would be interesting to further understand the contribution of the lg2 gene to host plant defense mechanisms under *Striga* infestation in the inbred lines studied.

The S5_215,584,703 is close (131.43 kb) to the gene “GRMZM2G103085-ereb13- AP2-EREBP”. EREBPs (also referred to as ethylene-responsive element-binding proteins) containing a single AP2 domain are involved in regulatory networks of response to hormones, pathogen attack, and environmental signals involving DREBs (dehydration responsive element binding proteins) and ERFs (ethylene responsive factors) [52, 53]. Interestingly, Li et al. [54] identified TaPARG-2A and TaPRG-2G in wheat, belonging to the AP2 subfamily of AP2/EREBP transcription factors, that are involved in regulation of diverse processes of plant development and stress response. Hirota et al. [55] reported that the AP2/EREBP gene *PUCHI* is required for morphogenesis in the early lateral root primordium of Arabidopsis. However, this putative gene has not been well investigated in maize and needs further studies for better understanding of its importance in *Striga* resistance.

Through this GWAS, we were able to detect reliable QTLs associated with maize plant defense mechanisms under *Striga* infestation. The information provided in this study would serve as the starting point for functional gene studies to clarify the genetic mechanisms underlying *Striga* resistance in tropical maize inbred lines. After validation, the significant loci identified in this study could be targets for breeders in marker-assisted selection to accelerate genetic enhancement of maize for *Striga* resistance in the tropics, particularly in the West and Central Africa sub-region.

## Conclusions and recommendations

To the best of our knowledge, the present study is the first report of genome-wide association analysis for *Striga* resistance in maize. Twenty-four SNPs were significantly associated with *Striga* adaptive traits in maize. The candidate putative genes GRMZM2G060216, GRMZM2G057243 and GRMZM2G164743, on chromosomes 3, 9 and 10, respectively, could be invaluable for the development of *Striga* resistant maize genotypes in SSA. Further studies, using different mapping populations, are urgently needed to validate the markers identified in the present study so that marker-assisted breeding for *Striga* resistance in tropical maize could be a reality and widely adopted in SSA where *Striga* is endemic.

## Methods

### Germplasm

A total of 132 early maturing tropical white maize inbred lines (comprising one hundred and twenty-six early maturing S_8_ inbred lines, five standard inbred testers and one inbred check) with combined resistance to *Striga hermonthica*, and tolerance to drought stress developed in the IITA maize improvement program were used in this study. Out of the 132 inbred lines, 126 were second generation lines extracted from six F_2_ populations derived from bi-parental crosses (TZE COMP 5-W DT C7 x TZEI 56, TZE COMP 5-W DT C7 x TZEI 87, TZE COMP 5-W DT C7 x TZEI 18, TZE COMP 5-W DT C7 x TZEI 31, TZE COMP 5-W DT C7 x TZEI 2, TZE COMP 5-W DT C7 x TZEI 65), which involved crosses among a broad based *Striga* resistant population (TZE COMP 5-W DT C7) and elite inbred lines from TZE-W Pop × 1368 STR (TZEI 87, TZEI 2), TZE-W Pop STR C_0_ (TZEI 65, TZEI 18 and TZEI 56), and TZE-W Pop x LD (TZEI 31). Of the five standard testers, inbreds TZEI 18, and TZEI 19 were derived from TZE-W Pop STR C_0_, inbred TZEI 31 from TZE-W Pop x LD, inbred TZEI 7 from WEC STR and inbred TZdEI 352 from TZE-W Pop STR.

The broad based *Striga* resistant population TZE COMP 5-W DT C7 from which the inbred lines evaluated in this study were derived, had gone through 7 cycles of recurrent selection for improved *Striga* resistance. TZE COMP 5-W DT C7 was crossed to the inbred lines TZEI 65, TZEI 18, TZEI 31, TZEI 87, TZEI 2 and TZEI 56 selected for drought tolerance to improve the population for drought tolerance. Following the introgression of the drought tolerance genes into the population, a program was initiated to extract inbred lines with combined *Striga* resistance and drought tolerance using repeated self-pollination and selection for *Striga* resistance and drought tolerance. After eight cycles of inbreeding, the inbred lines were evaluated under drought and *Striga* infestation. Based on the results of the evaluations, the S_8_ inbred lines used in the present study were selected.

### Phenotyping

The inbred lines were phenotyped for key agronomic traits across two environments under artificial *Striga* infestation and two *Striga*-free environments at Mokwa, Nigeria (9^0^18′N, 5^0^4′E, 457 m altitude, 1100 mm annual rainfall) during the rainy seasons of 2017 and 2018. The experimental design was 11 × 12 alpha lattice with two replications. Each experimental unit consisted of 3 m single-row plots, with a row spacing of 0.75 m and intra-row spacing of 0.4 m. The fields were injected with ethylene gas at about 10 days before planting, to stimulate suicidal germination of residual *Striga* seeds in the soil. The artificial *Striga* infestation at Mokwa was carried out as recommended by IITA-MIP [56]. *Striga* seeds collected from sorghum fields were stored for about 6 months to break seed dormancy and used for the infestation. Each hole in the *Striga* plot received about 5000 germinable seeds of *Striga* mixed with fine sand in the ratio 1:99. Fertilizer rate was reduced (30 kg N/ha, 30 kg each of P and K applied as NPK 15–15-15) and application was delayed till 3 weeks after planting to induce the production of strigolactones which stimulate good germination of the *Striga* seeds and the attachment of the *Striga* plants to the roots of host plants [57]. Under the artificial *Striga* infestation, data were collected on number of emerged *Striga* plants and host plant damage syndrome rating at 8 and 10 weeks after planting (WAP). The host plant damage syndrome rating was recorded on a scale of 1–9 (1 = normal plant growth, no visible symptoms, and 9 = complete scorching of all leaves, causing premature death or collapse of host plant and no ear formation). Under both *Striga*-infested and *Striga*-free environments, data were collected on the inbred lines for ear aspect, number of ears per plant and grain yield.

### Phenotypic data analysis

Analyses of variance (ANOVA) were performed across test environments for each experiment on plot mean basis for grain yield and other key agronomic traits with PROC GLM in SAS [58], using a RANDOM statement with TEST option. Location-year combinations were treated as environments. The IITA base index was used to identify *Striga* resistant and susceptible inbred lines under artificial *Striga* infestation [1]. The means of the selected traits were expressed in standard deviation units and the index scores were computed as: *I* = ((2 × YLD) + EPP - (SDR8 + SDR10) – 0.5(ESP8 + ESP10)), where YLD = grain yield of the *Striga*-infested plots, EPP is the number of ears at harvest in the *Striga* infested plots, SDR8 and SDR10 were *Striga* damage syndrome ratings at 8 and 10 WAP, and ESP8 and ESP10 were number of emerged *Striga* plants at 8 and 10 WAP. Broad sense heritability (H^2^) estimates were calculated from phenotypic variance (σ^2^_*p*_) and the genotypic variance (σ^2^_*g*_) [59]. Correlation analysis was done using the performance analytics package in R [60].

### Genotyping and quality control

Samples of leaves were taken in the field at 2 weeks after planting. The DArT protocol was used for genomic DNA extraction (www.diversityarrays.com/files/DArT_DNA_isolation.pdf). The quality and quantity of the DNA was ascertained by running the gDNA in a 1% agarose gel and measuring its concentration and purity in a NanoDrop 2000 spectrophotometer. The DNA samples were sent to the Integrated Genomic Service and Support (IGSS) genotyping platform, Nairobi, Kenya for genotyping. High-throughput genotyping was conducted in 96 plex DArTseq protocol as described previously [26]. Reads and tags found in each sequencing result were aligned to the *Zea mays* L. genome reference, version *AGPV3* (B73 Ref-Gen v4 assembly) [61], resulting in a raw dataset of 47,440 markers. The 47,440 DArTseq markers were filtered to eliminate SNPs with missing rate greater than 10%, heterozygosity greater than 20% and minor allele frequency (MAF) less than 5%. SNPs with unknown or multiple chromosomes locations were also eliminated. After quality filtering, a total of 7224 DArTseq markers distributed across the 10 maize chromosomes were used for the population structure, phylogenetic analysis and GWAS analyses.

### Population structure, linkage disequilibrium and marker-trait association analyses

An admixture model-based clustering method was used to infer population structure of the 132 genotypes using the software package STRUCTURE, version 2.3.4 [62]. The assumed number of subpopulations was simulated from *k* = 1 to *k* = 10 for an initial assessment of the most likely number of subpopulations; each K was run 10 times with 10,000 iterations of burn-in followed by 10,000 Markov chain Monte Carlo iterations and the ideal number of subpopulations (*K*) was found by examining the optimal ∆*K* value [19] in STRUCTURE Harvester [63]. In the model-based method, membership coefficients (*Q* values) for each inbred line were estimated to have its memberships in multiple subgroups. Inbred lines with membership probabilities ≥0.70 were assigned to the corresponding subgroup and lines with membership probabilities < 0.70 were assigned to a mixed subgroup. Linkage disequilibrium was determined using the squared allele frequency correlations R^2^ value from which the number of significant allele pairs (*P <* 0.01) was determined using 1000 permutations [64].. Association analysis between the SNPs and traits was performed using the mixed linear model (MLM) implemented in the GAPIT (Genetic Association and Prediction Integrated Tools) R package. The MLM adopted was proposed by Yu et al. [32] with each molecular marker considered a fixed effect and evaluated individually: Y = X_β_ + W_α_ + Q_v_ + Z_u_ + ε where *Y* is the observed vector of means; *β* is the fixed effect vector (*p* × 1) other than molecular markers effects and population structure; *α* is the fixed effect vector of the molecular markers; *ν* is the fixed effect vector from the population structure; *u* is the random effect vector from the polygenic background effect; *X*, *W*, and *Z* are the incidence matrixes from the associated *β*, *α*, *ν*, and *u* parameters; and *ε* is the residual effect vector. MLM in comparison with other models for detecting marker/trait associations such as the general linear model (GLM), could reduce the false-positive associations by controlling both types I and II errors [65, 66]. The Bonferroni correction showed a very stringent threshold. Consequently, a GWAS threshold of –log (p) = 4 was used to declare significant marker-trait associations, which was determined based on the Q-Q plots and distribution of *p*-values for all the traits [22, 35, 67]. To identify gene models for *Striga* resistance, the physical positions of the significant SNPs were compared with the MaizeGDB database according to version 4 (RefGen_v4) from the reference genome of the maize B73 inbred line, available at the MaizeGDB database. Zoom mapping was conducted on the chromosome where a significant SNP marker was identified and associated with a trait. The extent of local LD was evaluated for each selected significant SNP to determine the interval of each locus. The heatmap of regional LD was made with the LD heatmap package [68] for SNPs with a MAF greater than 0.05 within 500 kb downstream and upstream of the top associated SNP.

## Supplementary information

**Additional file 1: Figure S1**. Quality filtering of 44,470 markers among 132 maize inbred lines. Description of data: Quality filtering of 44,470 markers among 132 maize inbred lines that were used for population structure analysis and the GWAS.

**Additional file 2: Table S1**. Summary statistics of the 7224 filtered high-quality SNP markers. Summary statistics of the 7224 filtered high-quality SNP markers obtained from the total 44,470 markers.

**Additional file 3: Figure S2**. Genome-wide and chromosome-wide linkage disequilibrium (LD) decay plot. LD decay plot estimated based on pairwise squared allele frequency correlation coefficients (R^2^) among 7224 SNPs distributed across the 10 maize chromosomes. The values on the y-axis represent the squared correlation coefficient R^2^ and the x-axis represents the genetic distance in megabases (mb).

## Data Availability

The DArTseq datasets used in the present study have been deposited at the IITA repository. DOI: 10.25502/h7k9-3s55/d. Link to CKAN: http://data.iita.org/dataset/genotypic-data-for-maize-inbred-lines-for-diversity-studies-and-gwas.

## Abbreviations

QTL: Quantitative trait locus
ANOVA: Analysis of variance
chr: Chromosome
bp: Base pair
Kb: Kilo base pair
SNP: Single nucleotide polymorphism
GWAS: Genome-wide association study
SSA: Sub-Saharan Africa
MAF: Minor allele frequency
DArTseq: Diversity array technology sequencing
IITA: International Institute of Tropical Agriculture
MAS: Marker-assisted selection
LGS: Low germination stimulant
WAP: Weeks after planting
LD: Linkage disequilibrium
amt: Ammonium transporter
EREBPs: Ethylene-responsive element-binding proteins
DREBs: Dehydration responsive element binding proteins
MLM: Mixed linear model
GAPIT: Genetic association and prediction integrated tools
GLM: General linear model
