# Supplementary material for: Genome-wide association study of Striga resistance in early maturing white tropical maize inbred lines
Source: BMC Plant Biol. 2020 May 11;20:203. doi: 10.1186/s12870-020-02360-0 (PMC7212567; doi:10.1186/s12870-020-02360-0)
Supplement: Supplementary file 1 — Additional file 1: Figure S1. Quality filtering of 44,470 markers among 132 maize inbred lines. Description of data: Quality filtering of 44,470 markers among 132 maize inbred lines that were used for population structure analysis and the GWAS. [file 12870_2020_2360_MOESM1_ESM.docx]

**Additional file** **1:** **Figure S1.** Quality filtering of 44,470 markers among 132 maize inbred lines.
