# Supplementary figures and images for: Genome-wide association study of Striga resistance in early maturing white tropical maize inbred lines
Source: BMC Plant Biol. 2020 May 11;20:203. doi: 10.1186/s12870-020-02360-0 (PMC7212567; doi:10.1186/s12870-020-02360-0)

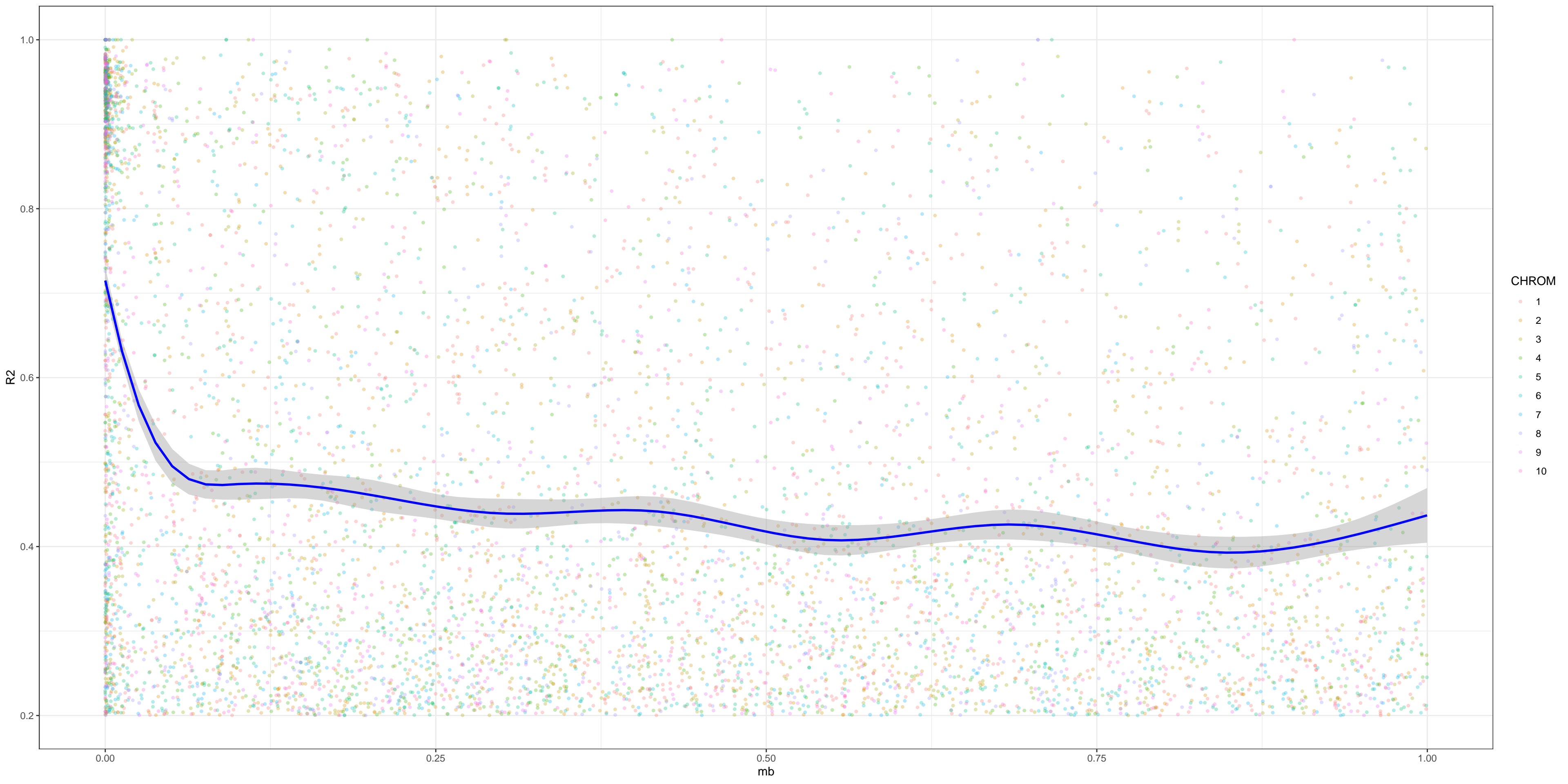

Supplement: Supplementary file 3 — Additional file 3: Figure S2. Genome-wide and chromosome-wide linkage disequilibrium (LD) decay plot. LD decay plot estimated based on pairwise squared allele frequency correlation coefficients (R2) among 7224 SNPs distributed across the 10 maize chromosomes. The values on the y-axis represent the squared correlation coefficient R2 and the x-axis represents the genetic distance in megabases (mb). [file 12870_2020_2360_MOESM3_ESM.pdf]
